# The Role of Language Proficiency in False Memory: A Mini Review

**DOI:** 10.3389/fpsyg.2021.659434

**Published:** 2021-04-09

**Authors:** Mar Suarez, Maria Soledad Beato

**Affiliations:** Faculty of Psychology, University of Salamanca, Salamanca, Spain

**Keywords:** false memories, false recognition, DRM paradigm, bilingualism, language proficiency

## Abstract

Memory errors and, specifically, false memories in the Deese/Roediger–McDermott paradigm have been extensively studied in the past decades. Most studies have investigated false memory in monolinguals’ native or first language (L1), but interest has also grown in examining false memories in participants’ second language (L2) with different proficiency levels. The main purpose of this manuscript is to review the current state of knowledge on the role of language proficiency on false memories when participants encode and retrieve information in the same language. To do so, a systematic literature search was conducted, and the available studies were reviewed. These studies differed in, for example, age, language proficiency, or material characteristics, including both high and low associative strength lists, and they reported different results. In this review, we attempted to make sense of the apparently contradictory results by carefully identifying participants’ language dominance and L2 proficiency. Specifically, the results indicated that, first, people are more prone to produce false memories in their dominant than in their non-dominant language. This result generalizes to lists with high and low associative strength, as well as to participants of different ages. Second, false memories do not differ between two languages when speakers are equally proficient in both languages. Finally, highly proficient L2 speakers produce more false memories in their L2 than speakers with lower L2 proficiency. The results of this review will be considered in the light of the theoretical frameworks of false memories and bilingual language processing.

## Introduction

In recent years, there has been considerable interest in investigating the malleable nature of human memory. Memory is a reconstructive process that is prone to errors (Kolodner, 1983) and this fact has implications in real-world settings such as eyewitness testimonies (Aizpurua et al., 2009; Havard and Memon, 2013; Loftus, 2018) or the clinical practice (Otgaar et al., 2017; Turk et al., 2020). Among all the potential memory errors, a substantial body of research has focused on understanding false memories, that is, memories for events that did not occur. Specifically, in this review we will focus on a particular type of false memory: the associative memory illusion (see Gallo, 2006, 2010 for review).

False memories have been mostly investigated in monolinguals of very diverse languages, such as Germanic languages (e.g., English, Stadler et al., 1999; Dutch, Van Damme and d’Ydewalle, 2009), Romance languages (e.g., Portuguese, Albuquerque, 2005; Spanish, Beato and Díez, 2011; French, Dubuisson et al., 2012) and Slavic languages (e.g., Polish, Ulatowska and Olszewska, 2013). Even non-Indo-European languages have been employed to study the associative memory illusion (e.g., Chinese, Chen et al., 2008; Japanese, Kawasaki and Yama, 2006), with a robust false memory effect in all of them. However, false memories have not only been studied in first languages (L1), but also in second languages (L2) with different proficiency levels (e.g., Anastasi et al., 2005; Arndt and Beato, 2017; Beato and Arndt, 2021).

It is important to note that language proficiency may vary with language usage and experience, and, therefore, it is not a constant feature. Bilingualism and monolingualism would be the two ends of a continuum with no clear division between them due to a lack of consensus on the definition of bilingualism (Edwards, 2004) and a high variability in its measurement (Surrain and Luk, 2019). In this review, our purpose is not to define bilingualism, but rather to study false memories across the proficiency continuum on participants with some knowledge of a second language.

Different questions have been investigated in the literature regarding false memories in various languages. First, some research has focused on false memories when languages are switched between encoding and retrieval (i.e., between-language false memory) (see Graves and Altarriba, 2014 for review). Second, another line of research has been interested in whether language and memory processes differ between bilinguals and monolinguals (e.g., Bialystok et al., 2020). Third, and the aim of this review, increasing interest has been centered on whether language proficiency influenced false memories when participants encode and retrieve information in the same language (i.e., within-language false memory). To this aim, we reviewed all the available articles investigating this topic that emerged from a systematic literature search. In particular, our goals were to (1) examine false recognition in the L1 versus L2, centering our attention on language dominance and L2 proficiency, and (2) discuss the findings in terms of the theoretical frameworks.

## False Memories: The DRM Paradigm

One of the most widely used paradigms to study false memories is the Deese/Roediger–McDermott (DRM) paradigm (Deese, 1959; Roediger and McDermott, 1995). In this paradigm participants study lists of words associated to a non-studied word (i.e., critical lure). For example, participants study the words *hot*, *ice*, *snow*, *warm*, *winter*, and *weather*, all of them associated to the critical lure *cold*, based on free association norms (e.g., Nelson et al., 1998). At the test, participants often falsely recall and/or recognize the critical lures as studied items.

The DRM paradigm has been extensively used to study the mechanisms underlying false memories by manipulating variables such as backward and forward associative strength (e.g., Brainerd and Wright, 2005; Arndt, 2012, 2015; Beato and Arndt, 2014, 2017), presentation rate (e.g., Seamon et al., 1998; Smith and Kimball, 2012; Sadler et al., 2018), number of words associated to the critical lure (e.g., Arndt, 2010; Flegal and Reuter-Lorenz, 2014), presentation modality (e.g., Mao et al., 2010; Boldini et al., 2013), retrieval time (e.g., Giammattei and Arndt, 2012; Carneiro et al., 2014), attentional demands (e.g., Pérez-Mata et al., 2002; Otgaar et al., 2012), distinctive encoding (e.g., Huff et al., 2015, 2020), warning instructions (e.g., Watson et al., 2004; Carneiro and Fernandez, 2010; Coane et al., 2016), identifiability of the critical lure (e.g., Neuschatz et al., 2003; Carneiro et al., 2009; Beato and Cadavid, 2016), or emotional valence (e.g., Bookbinder and Brainerd, 2016; Hellenthal et al., 2019; Chang et al., 2020), among many others. All these experimental manipulations confirmed the robustness of this paradigm to produce false memories.

Furthermore, the DRM paradigm has also been employed to study false memories in different clinical populations such as patients with schizophrenia (e.g., Bhatt et al., 2010; Favre et al., 2020), Alzheimer’s disease (e.g., Malone et al., 2019; Howe and Akhtar, 2020), or autism spectrum disorder (e.g., Wojcik et al., 2018). Additionally, it has been used throughout development in children (e.g., Carneiro et al., 2007; Brainerd et al., 2008; Knott et al., 2011), and older adults (e.g., McCabe et al., 2009; Devitt and Schacter, 2016).

Not only behavioral research has been conducted on false memories. Some efforts have also been made to identify the neural correlates of false memories (see Schacter and Slotnick, 2004 for review) using techniques such as functional magnetic resonance imaging (e.g., Abe et al., 2013), event-related potentials (e.g., Curran et al., 2001; Beato et al., 2012; Cadavid and Beato, 2016), near infrared spectroscopy (e.g., Kubota et al., 2006), positron emission tomography (Schacter et al., 1996), or transcranial direct current stimulation (e.g., Díez et al., 2017).

The two main theoretical explanations of the false memory effect in the DRM paradigm are the fuzzy-trace theory (FFT; Reyna and Brainerd, 1995; Brainerd and Reyna, 2002) and the activation-monitoring framework (AMF; Roediger et al., 2001) (see also global matching models, Arndt and Hirshman, 1998). According to FFT, two types of information are encoded during the study of the DRM lists: verbatim traces (i.e., perceptual features of the event) and gist traces (i.e., meaning-based information of the event). If gist memory traces are retrieved, false memories are more likely to occur because critical lures tend to match the meaning information extracted from the list. Therefore, the critical lure’s meaning would be familiar. That familiarity triggered by the critical lure might be countervailed by retrieving verbatim traces of the studied items, a process referred to as recollection rejection (Brainerd et al., 2003), that would reduce false memories. For its part, AMF suggests that false memories are produced by the combination of activation and monitoring processes. When a DRM list is studied these words are activated and the activation is spread throughout the semantic network to associatively related words, namely, the critical lure, increasing the likelihood to produce false memories. In order to counteract that activation, monitoring processes might be engaged. Monitoring processes are defined as decision processes that use different types of information to determine the source of the activation and so false memories can be reduced (Gallo, 2006). As will be discussed later, both theories (i.e., FFT and AMF) could potentially explain the results of the present review, but in slightly different ways.

## Two Languages in One Brain

A central question in bilingual research is how two languages are represented in one brain (Heredia and Brown, 2006). Various models have been developed to seek an answer (e.g., bilingual interactive activation model, Dijkstra and Van Heuven, 2002; inhibitory control model, Green, 1998; distributed feature model, Van Hell and De Groot, 1998; revised hierarchical model, Kroll and Stewart, 1994) and, despite differing in the exact nature of L1 and L2 representations, these models share a consensual view about two assumptions relevant for the present review. First, both languages access a shared conceptual system (Francis, 1999, 2020; Francis et al., 2019) and, second, associations between word forms and their concepts are stronger in L1 than in L2 (e.g., Gollan et al., 2008). To further elaborate on these ideas, the revised hierarchical model (RHM)^1^, referred above, will be considered.

The RHM assumes two different levels of representation, the lexical and conceptual level, with independent lexical representations for each language and a shared conceptual store. First, at the lexical level, although both languages are stored independently, they are interconnected with stronger connections from L2 to L1 than from L1 to L2 (Kroll et al., 2002). The explanation is found in the fact that the L2 is acquired by creating links between L2 words and the correspondent L1 translation at the lexical level, leading to stronger connection from L2 to L1. By contrast, links from L1 to L2 are weaker due to a lack of translation practice in that direction (Kroll and Stewart, 1994). Second, at the conceptual level, the links between words and concepts (i.e., conceptual links) are assumed to be stronger in L1 than L2. This means that the concept store is fully activated quicker from L1 than from L2 lexical representations. Nonetheless, once L2 learners become more proficient, the conceptual links from L2 words to the concepts become stronger (Perea et al., 2008).

Although the RHM interprets that the first language acquired (L1) is the dominant language, it is noteworthy that bilingual memory is a dynamic system influenced by language usage (Heredia and Altarriba, 2001). Thus, as Heredia (1997) suggested, the L1 might lose strength while the L2 might become the dominant language as a function of exposure, hence the L1 and L2 should be interpreted as the dominant and non-dominant language, respectively, disregarding which language was learned first.

## False Memories and Language Proficiency

In this review, we investigated the role of language proficiency in false memory when information was encoded and retrieved in L1 versus L2 (i.e., within-language conditions)^2^. To this end, we identified participants’ dominant and non-dominant language and their L2-proficiency level. This was crucial to understand and discuss the different results, but it was not an easy task. Specifically, regarding language dominance, sometimes it was difficult to identify which language was dominant (not necessarily the L1) based on the available information. Furthermore, regarding language proficiency, it was difficult to compare this variable across studies due to, first, different facets of bilingual experience being reported (e.g., usage or years of academic training; see Surrain and Luk, 2019). Second, there were differences in language proficiency (see Table 1), with some studies employing highly proficient bilinguals that used both languages in everyday life (e.g., Cabeza and Lennartson, 2005), while others included participants whose only L2 experience was in a classroom setting (e.g., Arndt and Beato, 2017). Third, although most of the studies included young adults (*M* = 24.04 years across experiments), children were also tested (Howe et al., 2008), possibly leading to age related differences in L1 and L2 proficiency.

**TABLE 1:** Summary of the reviewed studies on the role of language proficiency in false recognition.

Authors, year		Languages		Participants		Language proficiency and background	Results: false recognition		
			
		L1	L2	No.	Age (*M*)		L1	L2	Conclusion
Kawasaki-Miyaji et al., 2003		Japanese	English	74	University students (N/A)	L1: dominant language	0.71^1^	0.62^1^	L1 > L2^2^
						L2: 7 years of academic training			
						Participants lived in Japan			
Anastasi et al., 2005	Exp 1	Japanese, Spanish, German	English	12	University students (N/A)	L1: dominant language	0.55	0.49	L1 = L2^3^
						L2: 8.51 years of experience/academic training			
						Participants were exchange students in the United States			
	Exp 2	Spanish	English	22	Young adults (30.70)	L1: 75% at home, 45% at work, and 60% with friends	0.52	0.70	L1 < L2^3^
						L2 (dominant language): 50% at home, 75% at work, and 80% with friends^4^			
						Participants lived in the United States			
	Exp 3	Spanish	English	20	Young adults (29.70)	L1 (dominant language): 100% at home, work, and with friends	0.59	0.44	L1 > L2^3^
						L2: living in an L2 speaking country			
						Participants lived in the United States			
	Exp 4	English	Spanish	24	University students (N/A)	L1 (dominant language): 100% at home, work, and with friends	0.68	0.16	L1 > L2^3^
						L2: no formal instruction			
						Participants lived in the United States			
Cabeza and Lennartson, 2005		English	French	30	University students (N/A)	L1 and L2: high proficiency and used in everyday life	0.41	0.40	L1 = L2
						Participants lived in Canada			
Sahlin et al., 2005		English	Spanish	20	University students (20.00)	L1 (dominant language): proficiency self-report = 5/5	0.75^5^	0.62^5^	L1 > L2^3^
						L2: proficiency self-report = 4.55/5			
						Participants lived in the United States			
Howe et al., 2008		English	French	40	6 years old	L1 (children and adults): dominant language	0.74^1^	0.61^1^	L1 > L2
				32	8 years old	L2 (children): L2-immersion school.			
				30	12 years old	This was the only L2-speaking environment			
				20	20 years old	L2 (adults): participants were formally studying French			
						All lived in an L1 community in Canada			
Marmolejo et al., 2009		English	Spanish	60	University students (20.63)	L1 (dominant language): proficiency self-report = 9.35/10	0.80	0.73	L1 > L2^2^
						L2: proficiency self-report = 8.40/10			
						Participants lived in the United States			
Arndt and Beato, 2017	Exp 1	English	Spanish	28	University students (19.75)	L1: dominant language	0.29	0.15	L1 > L2
						L2: participants were formally studying a third-term Spanish course. Proficiency self-report = 6.32/10			
						Participants lived in the United States			
	Exp 2	Spanish	English	156	University students (22.48)	L1: dominant language	0.30	0.14	L1 > L2
						L2: studied on primary and secondary school. Proficiency self-report = 5.25/10			
						Participants lived in Spain			
	Exp 3	Spanish	English	52	University students (25.69)	L1: dominant language	0.35	0.18	L1 > L2
						L2: participants were formally studying English, 26 at elementary (low) level and 26 at advanced (high) level.		High: 0.22 Low: 0.13	High > Low
						Proficiency self-report: low = 4.31/10, high = 7/10			
						Participants lived in Spain			
Beato and Arndt, 2021	Exp 1	Spanish	English	90	University students (21.76)	L1: dominant language	0.33	0.21	L1 > L2
						L2: studied on primary and secondary school. Proficiency self-report = 6.02/10			
						Participants lived in Spain			
	Exp 2	Spanish	English	164	Young adults (29.69)	L1: dominant language	0.28	0.16	L1 > L2
						L2: participants were formally studying English, 58 at elementary (low) level, 59 intermediate (mid) level, and 47 advanced (high) level. Proficiency self-report: low = 5.36/10, mid = 6.54/10, high = 6.89/10 Participants lived in Spain		High: 0.19 Mid: 0.12 Low: 0.12	High > Mid High > Low Mid = Low

*False recognition rates are reported as mean proportions. L1, first language; L2, second language; N/A, not available. ^1^Means were provided by the first author in Kawasaki-Miyaji et al. (2003) and estimated form Figure 4 in Howe et al. (2008). ^2^The comparison was not tested statistically. ^3^Analyses were conducted on corrected scores in Anastasi et al. (2005) and sensitivity scores in Sahlin et al. (2005). ^4^Some participants reported using both languages in various environments. ^5^Only means for the first study-test trial are reported to be comparable to the other studies.*

Focusing now on the results, when comparing false memory in the L1 and L2, some studies found L1 > L2, others L1 < L2, or even, L1 = L2 (see Table 1). That is, although, to our knowledge, only eight works have investigated this topic, all possible results have been reported. However, as referred above, language dominance can make sense of the apparently contradictory results. In other words, if we compare false memory in the dominant and non-dominant languages, instead of considering the order of language acquisition (i.e., L1 versus L2), consistent conclusions can be drawn. To further elaborate on this idea, the three patterns of results observed in the reviewed studies will be explained below.

First, beginning with the most common result, the studies that found significantly higher false recognition in L1 than L2 (Anastasi et al., 2005, Experiments 3 and 4; Sahlin et al., 2005; Howe et al., 2008; Arndt and Beato, 2017; Beato and Arndt, 2021) reported that the dominant and non-dominant language were the L1 and L2, respectively. That is, false recognition was higher in the dominant than in the non-dominant language in these studies. Kawasaki-Miyaji et al. (2003) and Marmolejo et al. (2009) seemingly point in the same direction, with higher false recognition in L1 (dominant) than in L2 (non-dominant) (0.71 versus 0.62, and 0.80 versus 0.73, respectively), although they did not directly test this comparison statistically.

This pattern of results has also been found in 6-, 8-, and 12-year-old children (Howe et al., 2008). This study not only showed that false recognition increased with age in both languages, but also that all age groups were more likely to produce false recognition in L1 (dominant) than in L2 (non-dominant). Furthermore, the effect of language dominance on false memory was obtained in most of the studies using DRM lists strongly related to the critical lure, but Beato and Arndt (2021) found this effect with lists weakly related to the critical lure. Namely, higher false memories were reported in the dominant than in the non-dominant language in both adults and children, and with DRM lists that had high and low associative strength between the studied words and the critical lure.

Second, a study showed higher false recognition in L2 than L1 (Anastasi et al., 2005, Experiment 2). In this case, we can consider the L2 as the dominant language, since most of the participants frequently used this language at work (75%) and with friends (80%), and even half of participants used it at home. Therefore, here we can also conclude that false memories were higher in the dominant than in the non-dominant language.

Third, we identified two studies where false recognition was similar in the L1 and L2 (Anastasi et al., 2005, Experiment 1; Cabeza and Lennartson, 2005). In these cases, L1 and L2 proficiency seem to be similar. Specifically, Cabeza and Lennartson (2005) reported highly proficient speakers that used both languages in everyday life with no dominance difference specified. For their part, Anastasi et al.(2005, Experiment 1) included participants whose dominant language seems to be the L1, but it is also important to consider that participants had several years of L2 academic training, besides living in the L2-speaking country. As L2 acquisition and its associated brain changes are highly related to the amount of L2 immersion (Pliatsikas et al., 2017), it is reasonable to think that, in this case, participants could reach high levels of L1 and L2 proficiency at the moment of testing. Thus, in these two studies, we would expect false recognition not to differ significantly between L1 and L2 (since L1 and L2 proficiency would be similar), and this was exactly the result found in both experiments.

Lastly, two studies investigated false memories in participants that differed in L2 proficiency (Arndt and Beato, 2017; Beato and Arndt, 2021). Specifically, the authors found that greater language proficiency in the non-dominant language increased false memories. This result is in line with the above reported greater false recognition in the dominant (high proficiency, in these studies) than in the non-dominant (low proficiency, in these studies) language.

## Discussion

The reviewed studies suggest that, regardless of age and the associative strength of the lists, false memories are higher in participants’ dominant language than in their non-dominant one, just as false memories are greater in high than low L2 proficiency participants. Only when proficiency in the L1 and L2 is similar, false memories do not differ. These results could be accommodated by theoretical accounts from very different research areas, false memory and bilingual language processing.

Regarding the theoretical framework of the false memory effect, both the AMF and the FFT, mentioned above, could explain the current data despite claiming different mechanisms underlying the effect. On the one hand, according to FFT, the extraction of gist representations improves throughout development because participants become better in processing word meaning and connecting meaning across different words (Brainerd and Reyna, 2002). Given the parallel between how false memory differ across the proficiency continuum and the developmental trajectory of false memory (e.g., Carneiro and Fernandez, 2010; Arndt and Beato, 2017), this prediction can be used to explain the present results. That is, gist memory would be hindered when processing L2-words as participants have less experience, leading to a decrease in false memories. Along the same lines, gist memory improves when participants become more proficient, explaining why high L2 proficiency speakers show greater false memories than low L2 proficiency speakers. On the other hand, the activation processes referred by the AMF could explain the findings reviewed above by arguing that concepts are more automatically activated by the dominant than the non-dominant language, or even by high L2-proficiency participants. This activation spread throughout a well-organized network with strong connections to associatively related words (i.e., critical lure), which in turn would produce higher false memories in the dominant language than in the non-dominant one, as well as in high rather than low L2 proficiency speakers.

Within the bilingual language processing research, the greater false memories in the dominant than non-dominant language (e.g., Sahlin et al., 2005) could be accommodated by the RHM (e.g., Kroll and Stewart, 1994) since this model proposes stronger conceptual links in L1 than in L2. Furthermore, this model also assumes that the conceptual links in the L1 and L2 would have similar strength if proficiency in both languages is similar, predicting that false memories will not differ between L1 and L2 (e.g., Cabeza and Lennartson, 2005). Finally, as L2 proficiency increases, this theory suggests that the links between L2 words and their concepts strengthened, which predicts higher false memories for higher L2 proficiency participants (e.g., Arndt and Beato, 2017). With an increase in L2 proficiency as a function of language usage (Heredia, 1997), the L2 can even come to be the dominant language, in this case expecting higher false recognition in L2 than in L1 (e.g., Anastasi et al., 2005, Experiment 2).

In conclusion, this review has demonstrated that the DRM paradigm is useful to deepen our understanding of language and memory processes in speakers with knowledge of more than one language. Moreover, this review highlights the importance of language dominance to understand the production of false memories in the L1 and the L2. Therefore, we believe that it is crucial to assess language proficiency and exhaustively report participants’ language backgrounds on research that included more than one language. To do so some questionnaires have been created (e.g., Li et al., 2006; Marian et al., 2007; Luk and Bialystok, 2013; Anderson et al., 2018) that might be useful for future research. Additionally, as previous works showed that participants had far from perfect knowledge of L2 stimuli (Beato and Arndt, 2021), we encourage researchers to evaluate L2 word knowledge within future studies to assess the validity of alternative explanations for memory effects.

After reviewing the available articles investigating false memories in L1 versus L2, an issue that still seems unclear is whether participants translated L2 words during task performance (see Graves and Altarriba, 2014). Thus, further research could clarify this issue by manipulating the presentation rate of studied items or the time available during retrieval. Furthermore, researchers interested in measuring brain electrical activity need to describe the neural correlates of false recognition in the L1 and the L2 as, to our knowledge, no previous research has examined this matter. Additionally, it would be interesting to know whether false memories in the DRM paradigm differ between monolinguals and bilinguals. Although this issue is beyond the scope of this review, it would add valuable information to our understanding of language and memory processes in bilingual speakers.

## Author Contributions

Both authors listed have made a substantial, direct and intellectual contribution to the work, and approved it for publication.

## Conflict of Interest

The authors declare that the research was conducted in the absence of any commercial or financial relationships that could be construed as a potential conflict of interest.

## Footnotes

1 As some concerns have been raised regarding the RHM (e.g., Brysbaert and Duyck, 2010), see Kroll et al. (2010) for a critical review and assessment of this model.

2 Note that some reviewed studies also included other experimental conditions.
